# Mutagenesis Induced by Sub-Lethal Doses of Ciprofloxacin: Genotypic and Phenotypic Differences Between the *Pseudomonas aeruginosa* Strain PA14 and Clinical Isolates

**DOI:** 10.3389/fmicb.2019.01553

**Published:** 2019-07-10

**Authors:** Letícia Busato Migliorini, Holger Brüggemann, Romario Oliveira de Sales, Paula Célia Mariko Koga, Andrea Vieira de Souza, Marines Dalla Valle Martino, Rodrigo S. Galhardo, Patricia Severino

**Affiliations:** ^1^Hospital Israelita Albert Einstein, Albert Einstein Research and Education Institute, São Paulo, Brazil; ^2^Department of Biomedicine, Aarhus University, Aarhus, Denmark; ^3^Hospital Israelita Albert Einstein, Laboratorio Clinico, São Paulo, Brazil; ^4^Departamento de Microbiologia, Instituto de Ciências Biomédicas, Universidade de São Paulo, São Paulo, Brazil

**Keywords:** *Pseudomonas aeruginosa*, bacterial resistance, *umuDC*, ciprofloxacin, induced mutagenesis

## Abstract

Bacterial resistance is a severe threat to global public health. Exposure to sub-lethal concentrations has been considered a major driver of mutagenesis leading to antibiotic resistance in clinical settings. Ciprofloxacin is broadly used to treat infections caused by *Pseudomonas aeruginosa*, whereas increased mutagenesis induced by sub-lethal concentrations of ciprofloxacin has been reported for the reference strain, PAO1, *in vitro*. In this study we report increased mutagenesis induced by sub-lethal concentrations of ciprofloxacin for another reference strain, PA14-UCBPP, and lower mutagenesis for clinical isolates when compared to the reference strain. This unexpected result may be associated with missense mutations in *imuB* and *recX*, involved in adaptive responses, and the presence of Pyocin S2, which were found in all clinical isolates but not in the reference strain genome. The genetic differences between clinical isolates of *P. aeruginosa* and the reference PA14-UCBPP, often used to study *P. aeruginosa* phenotypes *in vitro*, may be involved in reduced mutagenesis under sub-lethal concentrations of CIP, a scenario that should be further explored for the understanding of bacterial fitness in hospital environments. Moreover, we highlight the presence of a complete *umuDC* operon in a *P. aeruginosa* clinical isolate. Even though the presence of *umuDC* did not contribute to a significant increase in mutagenesis, it highlights the dynamic exchange of genetic material between bacterial species in the hospital environment.

## Introduction

Bacterial resistance is a major threat to public health worldwide. Clinically relevant antimicrobial resistance has been associated with bacterial exposure to high concentrations of antibiotics selecting pre-existing resistant variants in the population (Andersson and Hughes, 2014). On the other hand, sub-lethal concentrations of antibiotics due to inappropriate use of antibiotics in human and veterinary medicine and agriculture may lead to changes in the transcription of bacterial genes which contribute to the adaptation and antimicrobial resistance (Kummerer, 2003; Gullberg et al., 2011; Laureti et al., 2013; Mathieu et al., 2016).

Among genes involved in adaptive responses, the SOS regulon and *umuDC*, are activated in response to DNA damage and encode error-prone polymerases involved in DNA translesion synthesis (TLS) and mutagenesis (Blazquez et al., 2006; Cirz et al., 2006; Friedberg et al., 2006; Baharoglu and Mazel, 2011; Thi et al., 2011). In *Escherichia coli*, damage-induced mutagenesis is heavily dependent on TLS polymerase V (PolV) encoded by *umuDC* genes (Elledge and Walker, 1983; Shinagawa et al., 1983; Reuven et al., 1999; Tang et al., 1999). Both, *in vitro* and *in vivo* studies have shown that the UmuDC complex is required for efficient TLS (Tippin et al., 2004). However, in *Pseudomonas* spp., *Mycobacterium tuberculosis*, and *Caulobacter crescentus*, that do not seem to carry the *umuDC* operon, *imuAB-dnaE2* have been implicated in SOS damage-induced mutagenesis (Galhardo et al., 2005; Cirz et al., 2006; Koorits et al., 2007; Warner et al., 2010). Both operons, *umuDC* and *imuAB-dnaE2*, are actively transcribed in TLS polymerase upon exposure to antibiotics such as ciprofloxacin (CIP). CIP inhibits the activity of DNA gyrase and topoisomerase II, triggering a genotoxic effect in bacteria (Drlica and Zhao, 1997; Blazquez et al., 2006; Cirz et al., 2006; O’Sullivan et al., 2008; Dörr et al., 2009). CIP is widely used in the treatment of infections caused by *P. aeruginosa* and varying concentration gradients may be observed in body compartments due to pharmacokinetics (Van Bambeke et al., 2005). For example, sub-lethal concentrations of antibiotics are frequently found in the lungs of patients with cystic fibrosis, where the increased viscosity of the lung mucus decreases the bioavailability of the drug (Moriarty et al., 2007; Ciofu et al., 2010; Jorgensen et al., 2013; Nair et al., 2013; Bhagirath et al., 2016). Additionally, increased mutagenesis induced by sub-minimum inhibitory concentration (MIC) of CIP has been reported, *in vitro*, for the reference strain PAO1 (Cirz et al., 2005; Torres-Barcelo et al., 2015; Wassermann et al., 2016; Valencia et al., 2017).

In a previous work by our group we reported clinical isolates of *P. aeruginosa* harboring *umuDC*-like sequences (Brüggemann et al., 2018). Here, we evaluate the mutagenesis induced by sub-MIC concentrations of CIP in clinical isolates, including a strain carrying the *umuDC* operon. In addition, we present a detailed description of the genomic location of these sequences, providing evidences of their insertion in *P. aeruginosa* genomes. This work aims to add to the knowledge on how genetic traits may impact mutagenesis in *P. aeruginosa* as well as differences between reference strains and clinical isolates regarding important aspects for human health.

## Methods

### Strains

The clinical isolates used herein have been previously described by Brüggemann et al. (2018). We selected three isolates from bronchoalveolar lavage (HIAE_PA11, HIAE_PA14 e HIAE_PA10) and three isolates from blood (HIAE_PA06, HIAE_PA12 and HIAE_PA15) (SisGen number A0DD69A) that were susceptible to CIP as determined by the Vitek system (Table 1); all these strains, except for HIAE_PA10, did not harbor *umuDC*-like sequences. We chose the reference strain PA14-UCBPP, which does not harbor *umuDC*, similarly to PAO1 (Cirz et al., 2006; Rybenkov, 2014), in order assess mutagenesis induced by sub-MIC of CIP in an additional reference strain and compare these results with clinical isolates. There was no clonal relatedness between clinical isolates, as previously determined by multi-locus sequence typing (MLST) analysis (Brüggemann et al., 2018), thus minimizing the hypothesis that the observed results are characteristic of a specific clone.

**TABLE 1:** Clinical isolates and reference strain used in this study.

**Strain**	**Year of isolation**	**Body site**	**Sequence type**	**GenBank accession numbers**	**Analysis performed**
PA14-UCBPP	1995	Wound	253	NC_008463	Genome analysis
HIAE_PA01	2011	Blood	455	QBET00000000	Genome analysis
HIAE_PA02	2012	Blood	381	QBES00000000	Genome analysis
HIAE_PA05	2013	Blood	498	QBER00000000	Genome analysis
HIAE_PA06	2014	Blood	231	QBEQ01000000	Mutagenesis, MIC of CIP and genome analysis
HIAE_PA07	2015	Blood	252	QBEP00000000	Genome analysis
HIAE_PA08	2015	Bronchoalveolar lavage	New	QBEO00000000	Genome analysis
HIAE_PA09	2015	Blood	245	QBEN00000000	Genome analysis
HIAE_PA10	2015	Bronchoalveolar lavage	2235	QBEM01000000	Mutagenesis, MIC of CIP and genome analysis
HIAE_PA11	2015	Bronchoalveolar lavage	244	QBEL01000000	Mutagenesis, MIC of CIP and genome analysis
HIAE_PA12	2015	Blood	10	QBEK01000000	Mutagenesis, MIC of CIP and genome analysis
HIAE_PA13	2015	Blood	244	QBEJ00000000	Genome analysis
HIAE_PA14	2015	Bronchoalveolar lavage	1290	QBEI01000000	Mutagenesis, MIC of CIP and genome analysis
HIAE_PA15	2016	Blood	1993	QBEH01000000	Mutagenesis, MIC of CIP and genome analysis
HIAE_PA16	1994	Bronchoalveolar lavage	606	QBEG00000000	Genome analysis
HIAE_PA17	1995	Tracheal secretion	235	QBEF00000000	Genome analysis
HIAE_PA18	1995	Bronchoalveolar lavage	348	QBEE00000000	Genome analysis
HIAE_PA19	1995	Tracheal secretion	235	QBED00000000	Genome analysis
HIAE_PA20	1997	Tracheal secretion	235	QBEC00000000	Genome analysis
HIAE_PA21	1998	Tracheal secretion	253	QBEB00000000	Genome analysis

*Sequence types were obtained by Multilocus Sequence Typing (MLST; Brüggemann et al., 2018).*

### Minimum Inhibitory Concentration

The MIC for CIP was estimated for all strains of *P. aeruginosa* using the broth microdilution method according to Clinical Laboratory Standards International (CLSI) recommendations (M100-S24) with the Mueller–Hinton culture medium (BD Difco). In this study the MIC is defined as the lowest antibiotic concentration in which there was no bacterial growth. The inoculum of each strain was diluted to 5 × 10^6^ colony forming units (CFUs)/mL and 10 μL of this dilution was dispensed into multiwell dishes (96 wells) containing 100 μl of the serial dilution of CIP. The multiwell dishes were incubated at 37°C for 18 h. The assays were carried out in triplicates and results were interpreted according to the (Clinical and Laboratory Standards Institute [CLSI], 2016).

### Mutation Frequency

The mutation frequency (Number of mutants/mL) was determined for all strains by the selection method based on the appearance of Fosfomycin (FOS) resistant mutants. In FOS resistant (FOS^R^) strains, mutations may comprise insertions, deletions and substitutions of nitrogenous bases that lead to loss of function of the *glpT* gene. This gene encodes a glycerol-3-phosphate permease, which is the only known protein responsible for the entry of FOS into *P. aeruginosa* (Castaneda-Garcia et al., 2009). The mutagenesis assay was performed as described by Blazquez et al. (2006). One single colony from each strain was inoculated into 5 ml Luria-Bertani (BD Difco) broth and incubated at 37°C overnight at 200 rpm. The culture was diluted 1: 1000 (Final volume = 50mL) in LB medium and incubated at 37°C, 200 rpm, until OD_600_ of 0.25. Then, 10 mL of this culture were divided into two flasks, one inoculum for control (spontaneous mutagenesis) and the other inoculum for treatment with CIP (induced mutagenesis). The antibiotic concentration used corresponds to 0.5 × MIC of CIP. Cultures were incubated overnight at 37°C at 200 rpm. Samples were then serially diluted and plated on solid LB medium without antibiotics to estimate the number of viable cells, and in LB medium containing FOS (128 μg/mL) and incubated at 37°C (Castaneda-Garcia et al., 2009). Subsequently, CFUs were counted for determination of mutant frequencies (mutants/viable cells). The relative increase between induced and spontaneous mutagenesis is represented here in the form of fold-change. Fifteen biological replicates were performed for each strain.

### Growth Curves

For bacterial growth curves a single colony was inoculated into liquid LB medium and incubated at 37°C, 200 rpm for 18 h. The next day, the cultures were diluted to an OD_600_ of 0.005, and measurements were collected every 30 min during exponential growth. For the growth curves in the presence of with sub-inhibitory concentrations of CIP, the same procedure was performed, however, upon reaching OD_600_ of 0.25, CIP was added to the medium at the respective concentration. The bacterial growth rates were estimated as previously described by Hall et al. (2014) using the formula ln (*N*_t_ − *N*_0_) = α(*t*_t_ − t_0_), where *N* = number of cells at time and α = first-order growth rate constant or the growth rate. In this case, α represent the slope of ln *N* versus *t* or ln OD versus *t* (Hall et al., 2014). Here, we calculated the growth rate in the exponential phase (120–270 min).

### Analysis of Genome Sequences

We selected genes previously described as overexpressed in *P. aeruginosa* under sub-inhibitory concentrations of CIP and involved in bacterial adaptive response under stress conditions (Blazquez et al., 2006; Cirz et al., 2006). The nucleotide coding sequences (CDSs) of *dinB*, *dnaE2*, *imuB*, *imuA*, *recA*, *lexA*, *recN*, and *recX* genes and their respective putative promoter regions were extracted from the genomes of the clinical isolates and compared to respective sequences from PA14-UCBPP by sequence alignment using the BLAST tool. Putative promoter regions were predicted using the software BPROM (Softberry Inc., Mount Kisco, NY, United States^1^). The upstream region (500 bp) of each gene was inspected and promoter regions were selected as described by Cebrián et al. (2014); in addition, LexA-binding sites, previously predicted by Cirz et al. (2006) were inspected in the strains. For the protein CDSs, to assess the possible impact of SNPs in protein function, the amino acid sequences were generated and aligned using the ClustalX2 program. Only common amino acid changes to all clinical isolates in relation to the PA14-UCBPP reference strain were considered for the purpose of this study.

We also performed protein analysis, evaluating common elements among clinical isolates that differed from the reference strain using data previously generated by our group (Brüggemann et al., 2018). All genomes of the strains used in this study were searched for *umuDC*-like sequences, using the studied *umuDC* of *E. coli* as query.

## Results

### Clinical Isolates Treated With Ciprofloxacin Showed Lower Survival and Mutagenesis Induced by Sub-Lethal Doses of CIP When Compared to PA14-UCBPP

**FIGURE 1:**
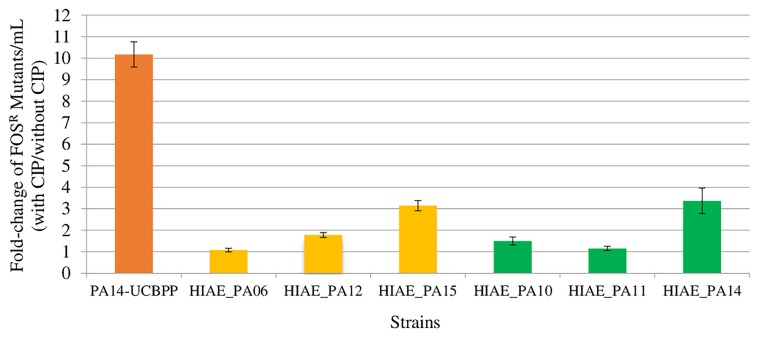
Induced mutagenesis (fold-change) following treatment with sub-inhibitory concentrations of CIP for PA14-UCBPP and six clinical isolates. Sub-inhibitory concentrations of CIP were 0.03 μg/mL for HIAE_PA11 and 0.06 μg/mL for the other strains. The orange bar corresponds to the reference strain PA14-UCBPP, the yellow bars correspond to the blood culture isolates and the green bars correspond to the bronchoalveolar lavage isolates. *N* = 15.

All strains used in this study were susceptible to CIP, with MIC, *in vitro*, of 0.125 μg/mL, with exception HIAE_PA11 with MIC of 0.06 μg/mL. Mutagenesis induced by sub-MIC CIP were lower in clinical isolates than in PA14-UCBPP (Figure 1). Sub-MIC CIP induced 10-fold mutagenesis in the reference line PA14-UCBPP, similar to that reported in the literature for PAO1 (Jorgensen et al., 2013; Torres-Barcelo et al., 2015; Wassermann et al., 2016; Valencia et al., 2017). By contrast, the highest induction in clinical isolates was approximately threefold in HIAE_PA14 and HIAE_PA15. This low mutagenesis rate was surprising, since mutagenesis in clinical strains could contribute to their adaptation in the hospital environment (Rodriguez-Rojas et al., 2013). To further investigate these findings, we determined the growth curve (optical density, OD_600nm_) for all strains in LB medium at 37°C, with and without CIP.

**FIGURE 2:**
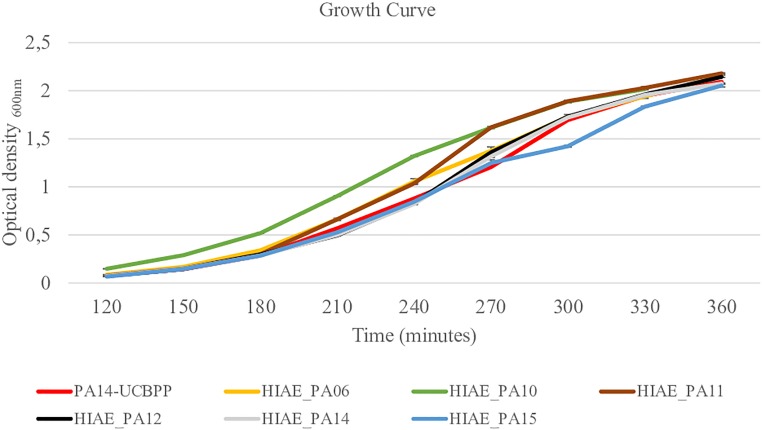
Growth curves for reference strain PA14-UCBPP and clinical isolates. *N* = 10.

**FIGURE 3:**
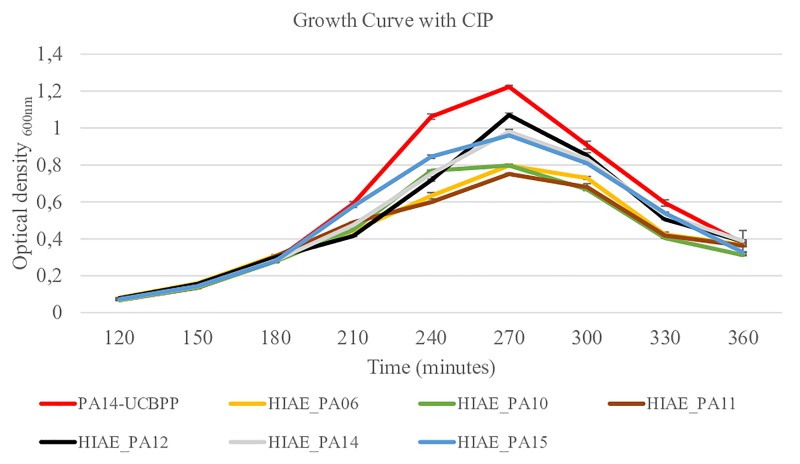
Growth curves for reference strain PA14-UCBPP and clinical isolates following treatment with sub-inhibitory concentrations of CIP. Sub-inhibitory concentrations of CIP were 0.03 μg/mL for HIAE_PA11 and 0.06 μg/mL for the other strains and CIP was added when OD_600_ reached 0.25 (at 180 min). *N* = 10.

Without treatment, the growth curves and the growth rate of clinical isolates and the reference strain were similar (Figure 2 and Table 2) and are similar to the growth curve presented for PAO1 in the literature: the lag phase takes approximately 2 h, followed by the log phase to about 4–6 h growth, and then steady state occurs (Lauridsen et al., 2017). However, after exposure to sub-MIC CIP, the PA14-UCBPP reference strain reached a higher OD than the clinical isolates, revealing a lower replication and/or higher mortality of clinical strains relative to the reference strain (Figure 3 and Table 2). In view of the results observed in this work, we conclude that exposure to CIP leads to different behavior patterns between clinical isolates and the reference strain.

**TABLE 2:** Growth rates for the clinical strains and PA14-UCBPP reference strain in exponential phase in the presence and absence of CIP.

	**PA14-UCBPP**	**HIAE_06**	**HIAE_10**	**HIAE_11**	**HIAE_12**	**HIAE_14**	**HIAE_15**
GR without CIP	0.018	0.018	0.019	0.020	0.020	0.019	0.019
GR with CIP	0.019	0.015	0.016	0.015	0.017	0.017	0.017

*GR: Growth rate.*

We then performed genome comparisons focused on genes previously associated with bacterial adaptive responses under stress conditions. Such genetic alterations could be implicated in the phenotype observed here (Figure 1).

### Sequence Analysis of Adaptive Response-Related Genes Reveals Differences in ImuB Protein Between PA14-UCBPP and Clinical Isolates

The sequence similarity in the adaptive response-related genes *dinB*, *dnaE2*, *imuB*, *imuA*, *recA*, *lexA*, *recN*, and *recX* between the reference strain PA14-UCBPP and clinical isolates ranged from 98 to 100%; the total number of SNPs is reported in Table 3. To assess the possible impact of these SNPs in mutagenesis following treatment at sub-MIC CIP, the amino acid sequences were compared (Table 4). Of the eight genes evaluated, six harbored silent mutations. The most affected CDS was *imuB*, with consequent 12 amino acid changes; RecX showed one amino acid exchange. The *imuB* gene is part of the *imuAB dnaE2* or *imuABC* operon, and it is present in bacteria that do not have *umuDC*, such as *Pseudomonas* spp., *M. tuberculosis* and *C. crescentus* (Boshoff et al., 2003; Galhardo et al., 2005; Koorits et al., 2007; Warner et al., 2010). In *E. coli*, *umuDC* encodes for PolV, an error-prone polymerase that participates in of TLS DNA (Kato and Shinoura, 1977; Caillet-Fauquet and Maenhaut-Michel, 1988). The inactivation of *umuDC* in *E. coli* and *imuAB dnaE2* in other bacteria are related to the decrease in damage-induced mutagenesis, and are therefore believed to be homologous in function and important for induced mutagenesis (Napolitano et al., 2000; Galhardo et al., 2005; Warner et al., 2010). We considered using protein homology to assess a possible impact of the alteration found in ImuB on its function, but no structures with sufficient homology were found in the Protein Data Bank^2^.

**TABLE 3:** Number of SNPs present in adaptive-response related genes of clinical isolates used in this study compared to PA14-UCBPP strain.

**PA14 genes**	**Gene**	**HIAE_6**	**HIAE_10**	**HIAE_11**	**HIAE_12**	**HIAE_14**	**HIAE_15**
PA14_52310	*dinB*	11	11	10	9	14	12
PA14_55610	*dnaE2*	32	36	41	23	34	39
PA14_55600	*imuB*	25	22	23	25	25	22
PA14_55590	*imuA*	9	11	22	6	12	11
PA14_17530	*recA*	6	6	5	10	4	6
PA14_25160	*lexA*	3	3	22	0	2	3
PA14_63010	*recN*	18	24	18	8	20	11
PA14_17540	*recX*	5	5	5	12	5	5

**TABLE 4:** Common amino acid changes to all clinical isolates in relation to the PA14-UCBPP in adaptive-response related genes.

*recA*	–
*lexA*	–
*imuA*	–
*imuB*	N21S; K147R; MRTTQ → LANSH (149 TO 153); NIS → GLA (155 TO 157); H166R; T304A
*dnaE2*	–
*dinB*	–
*recN*	–
*recX*	S149G

The *recX* gene is defined as *recA* co-regulator in *P. aeruginosa* and its inactivation is related to *recA* overexpression that may be lethal for the cell (Sano, 1993). Both, *recX* and *imuAB dnaE2* are activated by the SOS response and expressed in *P. aeruginosa* against DNA damage (Blazquez et al., 2006; Cirz et al., 2006). Although the consequences of the specific mutations in these genes remain unclear, it can be hypothesized that part of the survival phenotype observed here may be due to these alterations.

We then aimed to identify alteration in the predicted promoter regions that could have implications in gene expression regulation of these genes (Table 5). The nucleotide sequence for the predicted promoter region for *dinB* was identical for all clinical isolates except for one SNP (C to T) at position -52 relative to *dinB* transcription start codon when compared to the reference strain. For *lexA* we observed a SNP (C to T) at position -51 relative to *lexA* transcription start codon for strains HIAE_PA06, HIAE_PA10, HIAE_PA14 and HIAE_PA15. For strain HIAE_PA11 the sequence of nucleotides CGCCTC changed to GCGCAG at position -55 to -50 relative to *lexA* transcription start codon. However, all SNPs found in both *lexA* and *dinB* putative promoters were not within -10 or -35 regions, and should not, therefore, have an effect on RNA polymerase binding. The conserved -10 and -35 sequences are essential for the recognition and binding of σ_4_ and σ_2_ factors, respectively, being essential for gene expression (Arthur and Burgess, 1998; Campbell et al., 2003; Geszvain et al., 2004).

**TABLE 5:** SNPs identified in the putative promoter region of adaptive-response related genes of clinical isolates compared to PA14-UCBPP strain.

**Promoter of Gene**	**HIAE_6**	**HIAE_10**	**HIAE_11**	**HIAE_12**	**HIAE_14**	**HIAE_15**
*dinB*	C to T at position -52	C to T at position -52	C to T at position -52	C to T at position -52	C to T at position -52	C to T at position -52
*dnaE2*	–	–	–	–	–	–
*imuB*						
*imuA*						
*recA*	–	–	–	–	–	–
*recX*						
*lexA*	C to T at position -51	C to T at position -51	CGCCTC to GCGCAG at position -55 to -50	–	C to T at position -51	C to T at position -51
*recN*	–	–	–	–	–	–

*–, No SNP was found when the clinical isolate was compared to PA14-UCBPP strain. The SNPs position in putative promoter regions are relative to gene transcription start codon.*

We also investigated potential SNPs in the LexA-binding sites upstream of genes *dinB*, *imuA*, *imuB*, *dnaE2*, *recA*, *recX*, *lexA*, and *recN* that were previously predicted (Cirz et al., 2006). No SNPs could be found with one exception: strain HIAE_PA06 carries a SNP in an essential nucleotide position of the LexA-binding motif upstream of *recN*.

### Presence of *umuDC*-Like Sequences in Clinical Isolates of *P. aeruginosa*

**FIGURE 4:**
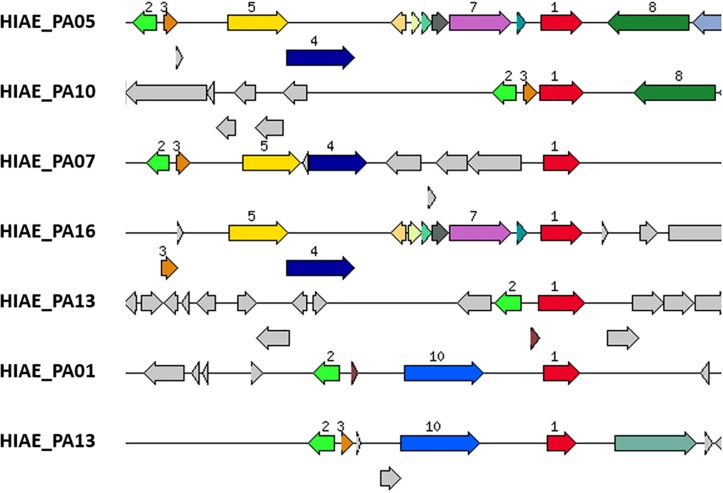
Genomic region of *umuC*-carrying strains of *P. aeruginosa.* The gene encoding an UmuC homolog is highlighted in red (number 1); it is present in six strains (strain HIAE_13 has two copies). UmuD-like sequences (orange, number 3) are present in close vicinity in five of these six strains. Only strain HIAE_10 carries a putative *umuCD* operon. In all six strains, the *umuCD*-containing region is strain-specific. Some adjacent genes are shared in two or more strains, encoding a Gifsy-2-prophage protein (light green, number 2), a recombinase/phage integrase (dark blue, number 4), a hypothetical protein (yellow, number 5), a DNA/RNA helicase (dark green, number 8), a mobile element protein (light blue, number 10). Interestingly, in two strains, a gene encoding a mercury resistance protein (violet, number 7) is closely associated with *umuC*.

Despite the previous notion that *umuDC* genes are absent from most *P. aeruginosa* strains (Abella et al., 2004; Erill et al., 2006; Koorits et al., 2007), we searched all clinical isolates of our cohort that were previously sequenced (Brüggemann et al., 2018) for homologs, using *E. coli*’s UmuC and UmuD amino acid sequences as query (Rybenkov, 2014). Interestingly, six out of 20 strains harbored sequences highly similar to UmuC (HIAE_PA01, HIAE_PA05, HIAE_PA07, HIAE_PA10, HIAE_PA13, and HIAE_PA16). Five of these strains also encoded a homolog of UmuD; the gene *umuD* was present in close vicinity to *umuC* in the five strains (Figure 4). Only in one strain, HIAE_PA10, the *umuDC* genes were organized in a putative operon. We wanted to determine the frequency of *umuC* and *umuD* in other strains of *P. aeruginosa*. Therefore, all strains with known completely closed genome sequences (*n* = 214) were searched, using *umuC* and *umuD* sequences from strain HIAE-PA10 as query. Out of 214 strains, 41 and 40 strains were *umuC-* and *umuD*-positive, respectively. Only 19 strains contained both, *umuC* and *umuD* (9% of all strains). This confirms that the presence of *umuDC* is rare among *P. aeruginosa* isolates.

Further inspection of the genomic region revealed that the *umuDC*-like sequences were part of the flexible genome, i.e., in genomic islands that are inserted into the core genome (Figure 4). Interestingly, the genomic region harboring *umuDC* varied strongly in all six strains, indicating that *umuDC* was acquired horizontally in different evolutionary events. Those events possibly included transduction, as judged from the presence of phage-like genes in the vicinity of *umuDC*. Another interesting observation was the association of the *umuC* gene with a gene encoding a putative mercury resistance protein in two strains (HIAE_PA05 and HIAE_PA16). In previous studies it was found that mercury resistance genes are located on mobile genetic elements such as plasmids and transposons in *Pseudomonas aeruginosa* and other *Pseudomonas* species (Hernández-Ramírez et al., 2018; Kottara et al., 2018). To know more about the occurrence of mercury resistance, we checked the 214 complete *P. aeruginosa* genomes: a gene for putative mercury resistance is found in 82 strains (38% of all strains). Interestingly, the mercury resistance gene was more often found in *umuCD*-positive strains [52% (10/19 strains)]. This underlines that *umuCD* is encoded within a flexible genomic region that can contain other fitness genes of the flexible gene pool.

### Presence of a Bacteriocin, Pyocin S2, in Clinical Isolates

It is interesting to note that RecA and LexA activities against DNA damage is not restricted to mutagenic response, but may also lead to the induction of phages and bacteriocins that decrease cell viability (Matsui et al., 1993; Fornelos et al., 2016). In order to investigate this additional aspect, we performed protein analysis comparing the clinical isolates and PA14-UCBPP: an antimicrobial bacteriocin (Pyocin S2) was present in all clinical isolates but absent in PA14-UCBPP. The antimicrobial protein Pyocin S2 is produced by *P. aeruginosa* for competition under stress conditions and may lead to cell death and, consequently, may interfere with mutagenesis (Okawa et al., 1973; Ohkawa et al., 1980; Michel-Briand and Baysse, 2002).

## Discussion

In view of the results observed in this work, we conclude that exposure to CIP leads to different behavioral patterns between clinical isolates and the reference strain PA14-UCBPP. Isolating bacteria from an infectious condition and subjecting it to laboratory monocultures may cause these strains to fail to express or lose genetic elements essential for survival *in vivo* and to express other genes necessary for *in vitro* survival (Ochman et al., 2000; Hayashi et al., 2001; Liang et al., 2001; Fux et al., 2005). The well-studied reference strains *P. aeruginosa* PAO1 (isolation year, 1955) and PA14-UCBPP (isolation year, 1995) have been sub-cultured for years since their first isolation, which has led to microevolution, possibly leading to new phenotypes (Holloway, 1955; Rahme et al., 1995; Klockgether et al., 2010; Cramer et al., 2011). In *P. aeruginosa*, these differences have been reported, for example, in the form of decreased ability to form biofilm by PAO1 and PA14-UCBPP relative to fresh clinical isolates, and in mutations that confer changes from non-mucoid to mucoid phenotypes, often found in *P. aeruginosa* isolates newly isolated but rarely detected under standard laboratory conditions (Schurr et al., 1996; Drenkard and Ausubel, 2002). In fact, the great genetic flexibility of *P. aeruginosa* represents a great concern regarding the reproducibility of the phenotypes observed in clinical strains compared with reference strains (Fux et al., 2005; Gaillard et al., 2011; Sidorenko et al., 2017). Also, the differences in phenotype between reference and clinical strains, and between *in vivo* and *in vitro* conditions, have been detailed for several bacteria, such as *E. coli*, *Staphylococcus aureus*, *Bordetella pertussis*, *Vibrio* sp., *Salmonella* sp. in which changes in biofilm production, virulence factors, growth rates, morphology and gene expression have already been observed (Lenski and Travisano, 1994; Edwards et al., 2002; Somerville et al., 2002; Reisner et al., 2006; Davidson et al., 2008; Gaillard et al., 2011; Ruwandeepika et al., 2011).

In our study, these differences are reported in phenotypic and genotypic characteristics. Both induced mutagenesis and replication rates and/or higher mortality of clinical strains as compared with the reference strain can be related to specific mutations in adaptive response-related genes such as *imuB* and *recX*, and the presence of Pyocin S2. The antimicrobial protein Pyocin S2 is produced by *P. aeruginosa* and may be advantageous under conditions when iron levels are low (Okawa et al., 1973; Ohkawa et al., 1980; Michel-Briand and Baysse, 2002). In fact, the levels of this bacteriocin are usually low, but under genotoxic stress it is induced. Similar to the SOS regulon, it is activated upon DNA damage leading to *recA* activation and PrtR repressor self-cleavage triggering *prtN* expression, necessary for the expression of the S, R, and F type pyocins (Matsui et al., 1993). Even though these mechanisms are activated by the same stimulus, and probably interact simultaneously with RecA co-protease, they trigger opposite responses. The activation of pyocins may lead to the reduction of resistance to genotoxic agents, such as antimicrobials, while the activation of the SOS response is related to the induction of low fidelity polymerases that participate in the TLS, subject to errors during replication, and therefore it is involved in mutagenic response in bacteria (Cirz et al., 2006; Penterman et al., 2014). Thus, Pyocin S2 present in all clinical genome strains but absent in PA14-UCBPP could play a role in viability and in the phenotype of mutagenesis under CIP sub-MIC observed for clinical isolates. The expression of this pyocin could lead to cell death and, consequently, no induction of mutagenesis. It is beyond the scope of this work to explore the function of SNPs found in *imuB* and *recX*, and the precise level of activity of Pyocin S2 in clinical isolates, but our results suggest that these genetic differences may be implicated in reduced mutagenesis under sub-MIC CIP and in survival rates.

Another important characteristic observed in our study is the presence of *umuDC*-like sequences in *P. aeruginosa* isolates. These sequences were found more frequently than anticipated, and in contrast with the two widely used laboratory strains PA14-UCBPP and PAO1. In fact, we are aware of only one study that has previously described an *umuD*-positive *P. aeruginosa* strain (Diaz-Magana et al., 2015); it was found that *umuD* was located on the conjugative plasmid pUM505, originally isolated from a clinical strain (Cervantes et al., 1990). Interestingly, it was shown that UmuD participates in the regulation of SOS gene expression; it was suggested that it functions as an anti-SOS effector (Diaz-Magana et al., 2015). Besides, the plasmid pUM505 also confers resistance to inorganic mercury. This suggests that metal resistance markers and *umuCD* could be physically linked, at least in some strains, on mobile genetic elements. This could be confirmed here for mercury resistance, as *umuCD*-positive strains are frequently also carrying mercury resistance determinants. The presence of *umuDC* and Pyocin S2 in a clinical isolate highlights the dynamics in the exchange of genetic elements between bacterial strains in a clinical setting and the phenotypic results reveals the need for caution when extrapolating results found in laboratory-adapted reference strains to clinical isolates, especially when it comes to new treatment options and therapeutic alternatives.

## Data Availability

The datasets generated for this study can be found in GenBank, NC_008463 QBEQ01000000, QBEM01000000, QBEL01000000, QBEK01000000, QBEI01000000, and QBEH01000000.

## Author Contributions

LM, RG, and PS conceived the study. LM carried out the experiments and analysis with support from RS and AS. LM and HB carried out the genome analysis. PK and MM were responsible for the clinical isolates maintenance and typing. LM, HB, and PS interpreted the results. LM and PS wrote the manuscript with contributions from HB and RG. PS supervised the project.

## Conflict of Interest Statement

The authors declare that the research was conducted in the absence of any commercial or financial relationships that could be construed as a potential conflict of interest.

## Footnotes

1 http://linux1.softberry.com

2 https://www.rcsb.org/
